# DNA methylome profiling reveals epigenetic regulation of lipoprotein-associated phospholipase A_2_ in human vulnerable atherosclerotic plaque

**DOI:** 10.1186/s13148-021-01152-z

**Published:** 2021-08-21

**Authors:** Jingjin Li, Xiaoping Zhang, Mengxi Yang, Hang Yang, Ning Xu, Xueqiang Fan, Gang Liu, Xintong Jiang, Jiasai Fan, Lifang Zhang, Hu Zhang, Ying Zhou, Rui Li, Si Gao, Jiangli Jin, Zening Jin, Jingang Zheng, Qiang Tu, Jingyi Ren

**Affiliations:** 1 Department of Cardiology, Beijing Tiantan Hospital of Capital Medical University, Beijing, China; 2 Beijing Anzhen Hospital of Capital Medical University and Beijing Institute of Heart Lung and Blood Vessel Diseases, Beijing, China; 3 Department of Cardiology, China-Japan Friendship Hospital, Beijing, China; 4 State Key Laboratory for Molecular and Developmental Biology, Institute of Genetics and Developmental Biology, Chinese Academy of Sciences, Beijing, China; 5 University of Chinese Academy of Sciences, Beijing, China; 6 Department of Medicine, Karolinska Institute, Stockholm, Sweden; 7 Department of Cardiovascular Surgery, China-Japan Friendship Hospital, Beijing, China; 8 Department of Cardiovascular Surgery, Peking University People’s Hospital, Beijing, China; 9 Department of Rheumatology and Immunology, Peking University People’s Hospital, Beijing, China; 10 Department of Neurology, China-Japan Friendship Hospital, Beijing, China; 11 Vascular Health Research Center of Peking University Health Science Center, Beijing, China

**Keywords:** DNA methylation, Atherosclerosis, Plaque vulnerability, Inflammation, *PLA2G7*, Lipoprotein-associated phospholipase A_2_

## Abstract

**Background:**

Atherosclerotic plaque vulnerability is a key feature of atheroprogression and precipitating acute cardiovascular events. Although the pivotal role of epigenetic regulation in atherosclerotic plaque destabilization is being recognized, the DNA methylation profile and its potential role in driving the progression and destabilization of atherosclerotic cardiovascular disease remains largely unknown. We conducted a genome-wide analysis to identify differentially methylated genes in vulnerable and non-vulnerable atherosclerotic lesions to understand more about pathogenesis.

**Results:**

We compared genome-wide DNA methylation profiling between carotid artery plaques of patients with clinically symptomatic (recent stroke or transient ischemic attack) and asymptomatic disease (no recent stroke) using Infinium Methylation BeadChip arrays, which revealed 90,368 differentially methylated sites (FDR < 0.05, |delta beta|> 0.03) corresponding to 14,657 annotated genes. Among these genomic sites, 30% were located at the promoter regions and 14% in the CpG islands, according to genomic loci and genomic proximity to the CpG islands, respectively. Moreover, 67% displayed hypomethylation in symptomatic plaques, and the differentially hypomethylated genes were found to be involved in various aspects of inflammation. Subsequently, we focus on CpG islands and revealed 14,596 differentially methylated sites (|delta beta|> 0.1) located at the promoter regions of 7048 genes. Integrated analysis of methylation and gene expression profiles identified that 107 genes were hypomethylated in symptomatic plaques and showed elevated expression levels in both advanced plaques and ruptured plaques. The imprinted gene *PLA2G7*, which encodes lipoprotein-associated phospholipase A_2_ (Lp-PLA_2_), was one of the top hypomethylated genes with an increased expression upon inflammation. Further, the hypomethylated CpG site at the promoter region of *PLA2G7* was identified as cg11874627, demethylation of which led to increased binding of Sp3 and expression of Lp-PLA_2_ through bisulfate sequencing, chromatin immunoprecipitation assay and enzyme-linked immunosorbent assay. These effects were further enhanced by deacetylase.

**Conclusion:**

Extensive DNA methylation modifications serve as a new and critical layer of biological regulation that contributes to atheroprogression and destabilization via inflammatory processes. Revelation of this hitherto unknown epigenetic regulatory mechanism could rejuvenate the prospects of Lp-PLA_2_ as a therapeutic target to stabilize the atherosclerotic plaque and reduce clinical sequelae.

**Supplementary Information:**

The online version contains supplementary material available at 10.1186/s13148-021-01152-z.

## Introduction

The atherosclerotic cardiovascular disease (ASCVD) remains to be the primary cause of morbidity and mortality worldwide, despite many and continued advances in medical therapies [1]. Clinically, atherosclerosis manifests itself mainly at the advanced stages when the plaques undergo rupture or erosion with superimposed thrombosis, which usually leads to acute ischemic events, including myocardial infarction (MI) and ischemic stroke [2]. Thus, sensitive detection of plaque vulnerability and pertinent intervention at early stages are therefore the major goals of treating acute cardiovascular events and its consequences.

Recent studies have been focusing on the potentially vulnerable plaque that exhibits remarkable plasticity and can change status in response to local environmental cues [3]. The regulation of these processes at the level of epigenetics remains largely unexplored. Epigenetic alteration results in phenotypic change without affecting the genomic DNA sequence, which is a regulation layer sensitive to environmental change and may provide a link between environment and gene expression [4]. Many risk factors of atherosclerosis, including hypertension, cholesterol homeostasis, and smoking, have been shown to impact epigenetic regulation [5–7]. Among the epigenetic modifications, DNA methylation has been demonstrated to alter the expression of several individual genes involved in plaque progression and vulnerability (e.g. *AIRE1*, *ALOX12*, and *IL-6*, etc.) and act as a critical component of the epigenetic regulation [8, 9]. However, a global view of DNA methylation in vulnerable and non-vulnerable atherosclerotic lesions is lacking.

**Fig. 1:**
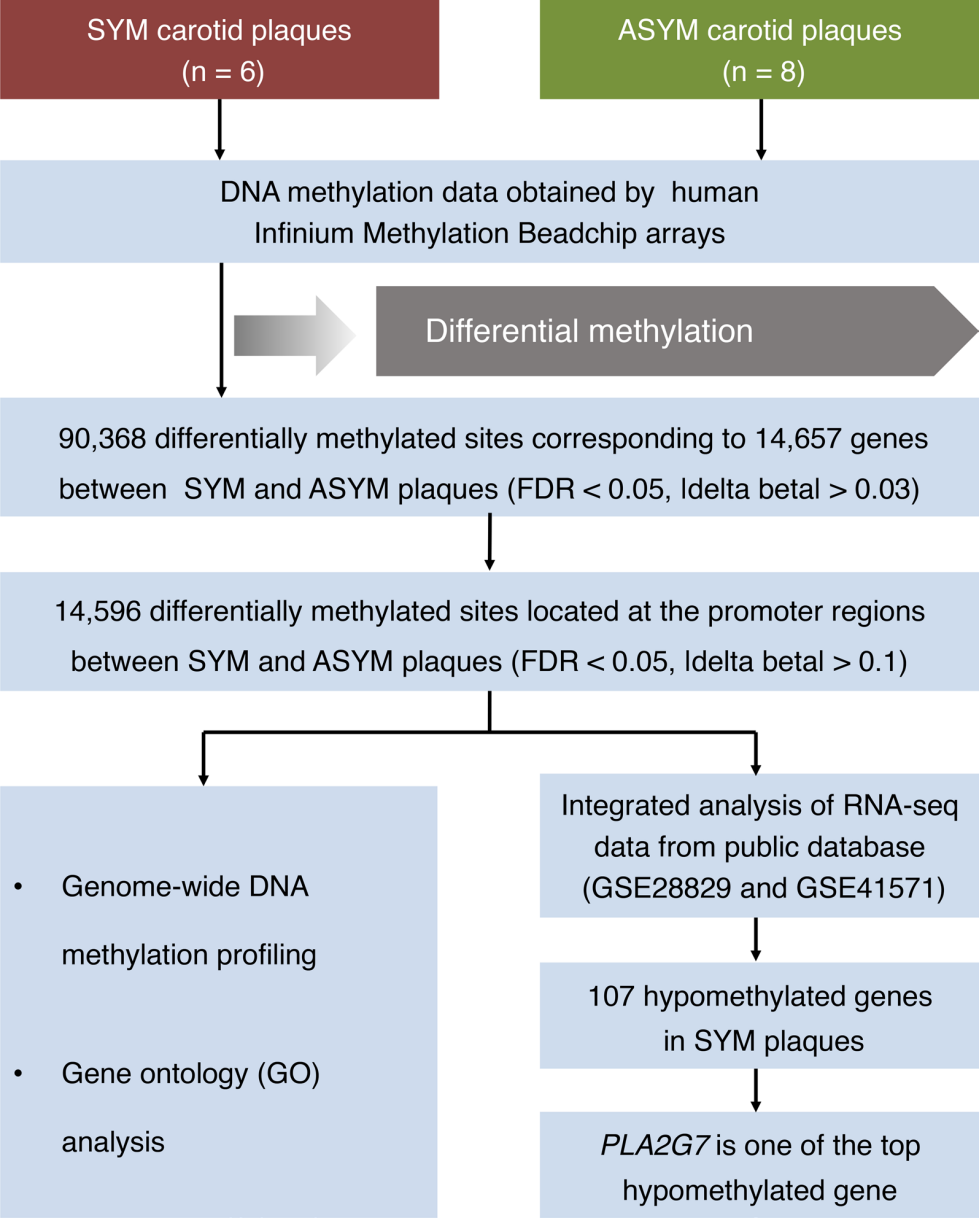
Schematic workflow of sample origin and the techniques used in atherosclerotic plaques. SYM, symptomatic; and ASYM, asymptomatic

Thus, to gain insights into the role of epigenetic regulation in the pathogenesis of atherosclerotic lesion progression and destabilization, we collected vulnerable and non-vulnerable plaque specimens from patients undergoing carotid endarterectomies (CEAs) and human left internal mammary arteries (LIMAs) tissue samples from patients undergoing coronary artery bypass  graft (CABG) procedure. A schematic flowchart  describing the source of the samples and the techniques used is shown Fig. 1 and Additional file 1. Profiling of the DNA methylome was done on these plaques to identify 90,368 differentially methylated sites corresponding to 14,657 genes in vulnerable plaques compared with non-vulnerable plaques. Interestingly, 67% of the affected sites were hypomethylated in vulnerable plaques, and the related genes were significantly enriched in pathways involved in the inflammatory response. Subsequently, we focus on genes differentially hypomethylated at promoter regions and revealed 14,596 differentially methylated sites (|delta beta|> 0.1) corresponding to 7048 genes. Integrated analysis of methylation and gene expression profiles identified that 107 genes were hypomethylated in symptomatic plaques and showed elevated expression levels in both advanced plaques and ruptured plaques. In particular, we identified *PLA2G7*, which encodes the lipoprotein-associated phospholipase A_2_ (Lp-PLA_2_), as a top hypomethylated gene with an increased expression upon inflammation. We found that the hypomethylation of the CpG islands at the promoter of the human *PLA2G7* gene altered the binding of transcription factor Sp3 to it, contributing to the upregulation of this gene.

## Results

### DNA methylation profiling identifies epigenetic signatures of plaque vulnerability

Human symptomatic (SYM) (*n* = 6) and asymptomatic (ASYM) plaques (*n* = 8) and human LIMA tissue samples (*n* = 4) were compared. There was no significant difference in baseline characteristics of the patients for SYM and ASYM plaques and normal arteries (Table 1).

**Table 1:** Characteristics of patients undergoing CEA depending on occurrence of symptoms and undergoing CABG

	LIMA (*n* = 4)	ASYM (*n* = 8)	SYM (*n* = 6)	*p* value
Age (years)	68.8 ± 5.4	70.3 ± 7.3	71.8 ± 9.0	0.818
Males (%)	4 (100.0)	8 (100.0)	6 (100.0)	1.000
Smokers (%)	2 (50.0)	6 (75.0)	3 (50.0)	0.569
CHD (%)	4 (100.0)	5 (62.5)	4 (66.7)	0.529
Hypertension (%)	4 (100.0)	4 (50.0)	5 (83.0)	0.207
Diabetes (%)	2 (25.0)	2 (25.0)	4 (66.7)	0.344
Fasting lipoproteins, (mmol/L)				
Total cholesterol	3.5 ± 1.2	4.9 ± 1.3	4.1 ± 1.7	0.266
LDL cholesterol	2.2 ± 1.3	2.8 ± 1.2	2.6 ± 1.9	0.469
HDL cholesterol	0.7 ± 0.2	1.4 ± 0.4	1.5 ± 0.6	0.055
Triglycerides	1.4 ± 0.2	1.9 ± 0.8	1.5 ± 1.0	0.568
Aspirin (%)	4 (100.0)	8 (100.0)	6 (100.0)	1.000
Statin (%)	4 (100.0)	4 (50.0)	5 (83.3)	0.207

*CEA* carotid endarterectomy, *CABG* coronary artery bypass  graft, *LIMA* left internal mammary artery, *SYM* symptomatic, *ASYM* asymptomatic, *LDL* low density lipoprotein, *HDL* high density lipoprotein

**Fig. 2:**
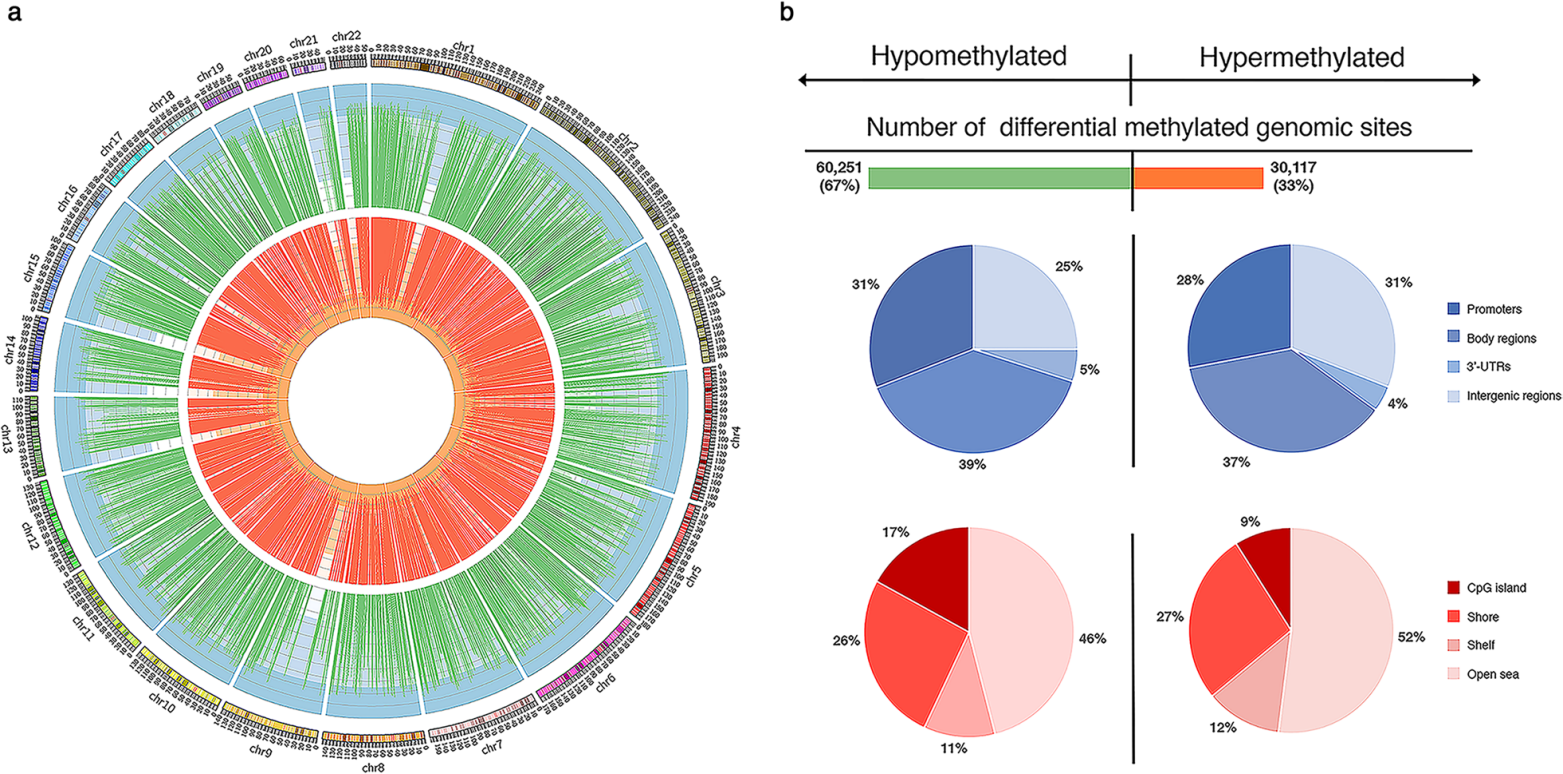
The genome-wide signature of DNA methylome in SYM and ASYM plaques. **a** Circos graph of DNA methylation levels in atherosclerotic SYM plaques (*n* = 6) in comparison with ASYM plaques (*n* = 8). The outer circle displays human chromosomes with scale at 1 MB bins. The hypomethylation probes are displayed as green lines, and hypermethylation probes are displayed as red lines. The height of the blue background indicates the scale of beta-scores in DNA methylation assays. **b** Hypo- and hypermethylated in SYM plaques compared with ASYM plaques are displayed on the left and right panels, respectively. The blue pie charts display the relative distribution of differential hypo- and hyper-methylated regions in promoters, gene bodies, 3'-UTRs, and intergenic regions. The red pie charts display the relative distribution of differential hypo-and hypermethylated regions in CpG islands, shore, shelf, and open sea regions. SYM, symptomatic; and ASYM, asymptomatic

We compared the DNA methylation profiles of SYM (*n* = 6) and ASYM plaques (*n* = 8) to understand the role of epigenetic regulation in plaque destabilization. We identified 90,368 differentially methylated sites (false discovery rate [FDR] < 0.05, |delta beta|> 0.03) corresponding to 14,657 genes from the total 450,000 probes (Fig. 2a; Additional file 2).

We classified these differential methylated sites into four categories according to their genomic loci: 30% of them were located in promoters (within 1500 bp upstream of the transcription start sites, or in the 5'- untranslated regions (UTRs), or in the first exon), 38% were in the gene body regions, 5% were in the 3'-UTRs, and the rest 27% were in the intergenic regions. Notably, among all the differential methylated sites, 67% (60,251/90,368) displayed hypomethylation in SYM plaques (Fig. 2b). In particular, 31% of these hypomethylated sites were located in promoters, 39% in the gene body regions, 5% in the 3'-UTRs, and 25% in the intergenic regions. The genomic loci of hypermethylated sites exhibited similar distribution as the hypomethylated sites (Fig. 2b).

Moreover, according to their genomic proximity to the CpG islands, we identified 14% of the differential methylated sites in the CpG islands, 27% were in the shore regions (up to 2 kb from CpG island), 12% in the shelf regions (from 2 to 4 kb from CpG island), and 48% were in the open sea regions (the rest of the genome). For the hypomethylated sites in SYM plaques, 17% were found in the CpG islands, 26% in the shore regions, 11% in the shelf regions, and 46% in the open sea regions (Fig. 2b). The corresponding percentages for the hypermethylated sites in SYM plaques were 12%, 27%, 9%, and 52%, respectively (Fig. 2b).

Also, we compared all atherosclerotic plaques (*n* = 14) with LIMAs (*n* = 4). The percentage of differentially hypomethylated sites was 49% (Additional file 3), which was significantly lower than that in SYM (67%). These results suggested that DNA hypomethylation could be a characteristic feature of vulnerable plaques. The distribution of differential hypomethylation was 29% in promoters, 34% in the gene body regions, 3% in the 3'-UTRs, and 34% in the intergenic regions. According to their proximity to the CpG islands, we identified 9% hypomethylated sites in the CpG islands, 16% in the shore regions, 21% in the shelf regions, and 54% in the open sea regions. Together, we identified a major shift of DNA methylome vulnerable plaques in humans compared with non-vulnerable plaques as well as lesion-free vessels, suggesting a potential pathological role of epigenetic regulation in plaque destabilization.

### Key biological processes involved in vulnerable plaque are subjected to the regulation of DNA methylation

**Fig. 3:**
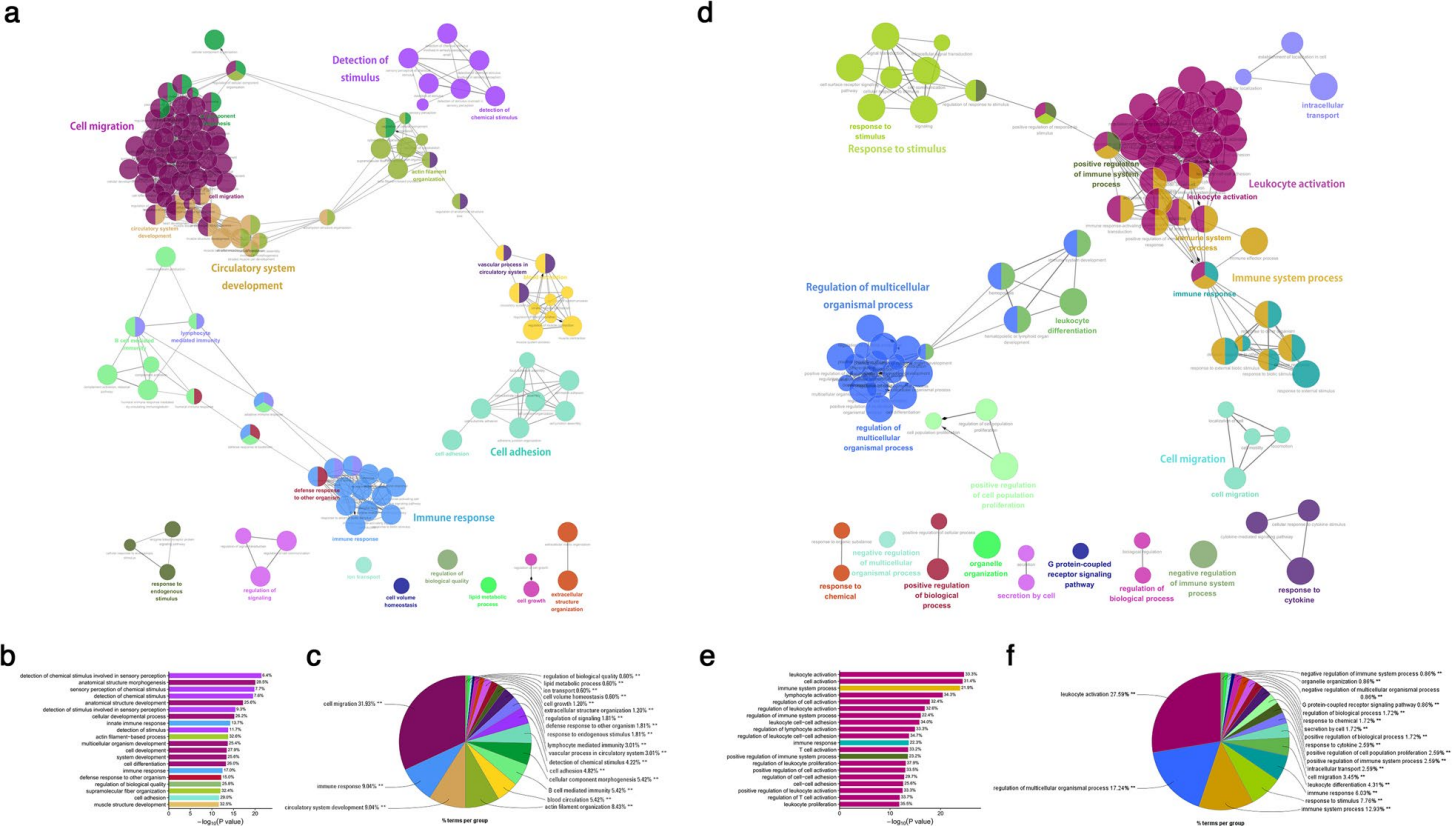
The biological function of the genes differentially methylated in SYM and ASYM plaques. **a** and **d** GO analysis of hypomethylated and hypermethylated genes in SYM compared with ASYM plaques using ClueGO and DAVID (*n* = 6 for SYM plaques and *n* = 8 for ASYM plaques). Nodes are linked based on their kappa score levels (> 0.03). The names of each group are labeled. The node size demarcates the enrichment significance of each term. Functionally related groups are partially overlapped. **b** and **e** Histogram of the most enriched biological function-specific terms of hypomethylated and hypermethylated genes. The length of the bar is the percentage of hypomethylated genes in each functional term (GO database), and the corresponding gene number is presented beside the bar. Those terms are sorted in the order of *p* value. **c** and **f** Overview pie-chart of the hypomethylated and hypermethylated genes show enriched functional groups, the percentage of certain groups were obtained from the GO analysis. SYM, symptomatic; ASYM, asymptomatic; GO, gene ontology; and DAVID, the database for annotation, visualization and integrated discovery

To understand the pathophysiological role of the differentially methylated genes between SYM and ASYM plaques, we focused on protein-coding genes differentially hypomethylated at promoter regions. Promoter regions show few dynamics across healthy tissues, whereas aberrant promoter methylation has been observed in variable diseases [10]. We revealed 14,596 differentially methylated sites (|delta beta|> 0.1) located at the promoter regions of 7048 genes, which included 9863 hypomethylated (corresponding to 4695 genes) and 4733 hypermethylated (corresponding to 2353 genes) sites. We performed gene ontology (GO) analysis for these genes by using ClueGO and database for annotation, visualization, and integrated discovery (DAVID) (Fig. 3; Additional file 4; Additional file 5). We found that the genes with hypomethylated promoters were mainly involved in biological processes, including cell migration, immune system process, cell adhesion, B cell-mediated immunity, and lymphocyte-mediated immunity (Fig. 3a–c). The genes with hypermethylated promoters in SYM plaques exerted functions, such as leukocyte activation, immune system process, response to stimulus, and leukocyte differentiation (Fig. 3d–f). This analysis suggested that the expression of inflammation and immunity-related genes were subjected to epigenetic regulation, which may play an essential role in the pathogenesis of plaque destabilization and progression.

Furthermore, we examined epigenetic changes in several inflammation and immune related biological process. Among genes with hypomethylated promoters, 531, 506, 232, and 151 genes involved in cell migration, cell adhesion, regulation of immune response and activation of immune response, respectively. Meanwhile, among genes with hypermethylated promoters, 178, 124, and 112 genes involved in regulation of immune system process, cell activation and leukocyte activation, respectively (Additional file 4; Additional file 5). These results indicated that a complex inflammatory network was involved in the pathogenesis of plaque destabilization.

Together, our data suggested that DNA methylation played an important role in regulating inflammation and immune response during plaque formation and progression (Fig. 3; Additional file 6). In addition, compared with plaque formation (atherosclerotic plaques vs. lesion-free arterial vessels), more genomic sites were found to be hypomethylated during plaque destabilization (SYM plaques vs. ASYM plaques), suggesting that reduced DNA methylation may contribute to the development of vulnerable plaques via inflammatory and immune processes.

### Integrated analysis of DNA methylation and transcription profiles in SYM and ASYM plaques

**Fig. 4:**
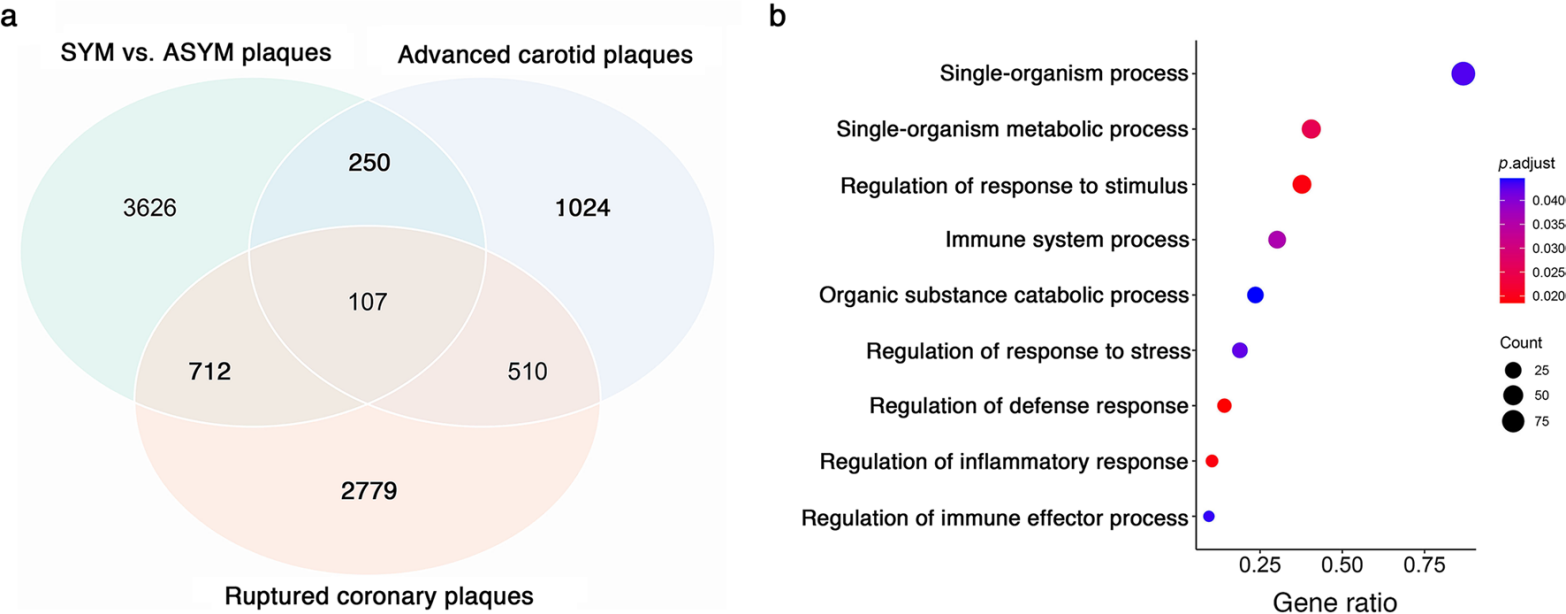
Integrated analysis of methylation and transcriptomic profiles of human atherosclerotic plaques. **a** Venn diagram depicting the number of genes hypomethylated in SYM compared with ASYM plaques (green), upregulated in advanced compared with early human carotid plaques (blue), and upregulated in macrophages from rupture compared with stable human coronary plaques (red). **b** GO biological process terms of genes enriched biological process. The size of the spots indicates the number of genes in the corresponding functional term. SYM, symptomatic; ASYM, asymptomatic; and GO, gene ontology

SYM plaques showed two-times more hypomethylated DNA sites than the hypermethylated ones (60,251 vs. 30,117). To probe the potential impact of DNA hypomethylation on gene expression in SYM plaques, we performed an integrated analysis of DNA methylome and transcriptome in human vulnerable atherosclerotic plaques samples. DNA hypomethylation at promoter region has been linked to increased gene expression [10], and macrophages, as modulators of inflammation, have a major role in plaque progression, leading to plaque disruption. Hence, we compared the hypomethylated genes in SYM vs. ASYM plaques with the genes upregulated in human advanced vs. early carotid plaques (GSE28829) [11], as well as with the genes with increased expression in macrophages from human rupture vs. stable coronary plaques (GSE41571) (Fig. 4a; Additional file 7) [12]. The results broadly matched the findings in methylation profiles at the gene function level, and the specific expression of genes hypomethylated in the SYM plaques was upregulated in both conditions. We identified 107 genes that were hypomethylated in SYM plaques and showed elevated expression levels in both advanced plaques and ruptured plaques. GO analysis revealed that these genes were primarily involved in processes related to inflammation (Fig. 4b; Additional file 8).

### *PLA2G7* is one of the top hypomethylated gene and a potential therapeutic target

**Fig. 5:**
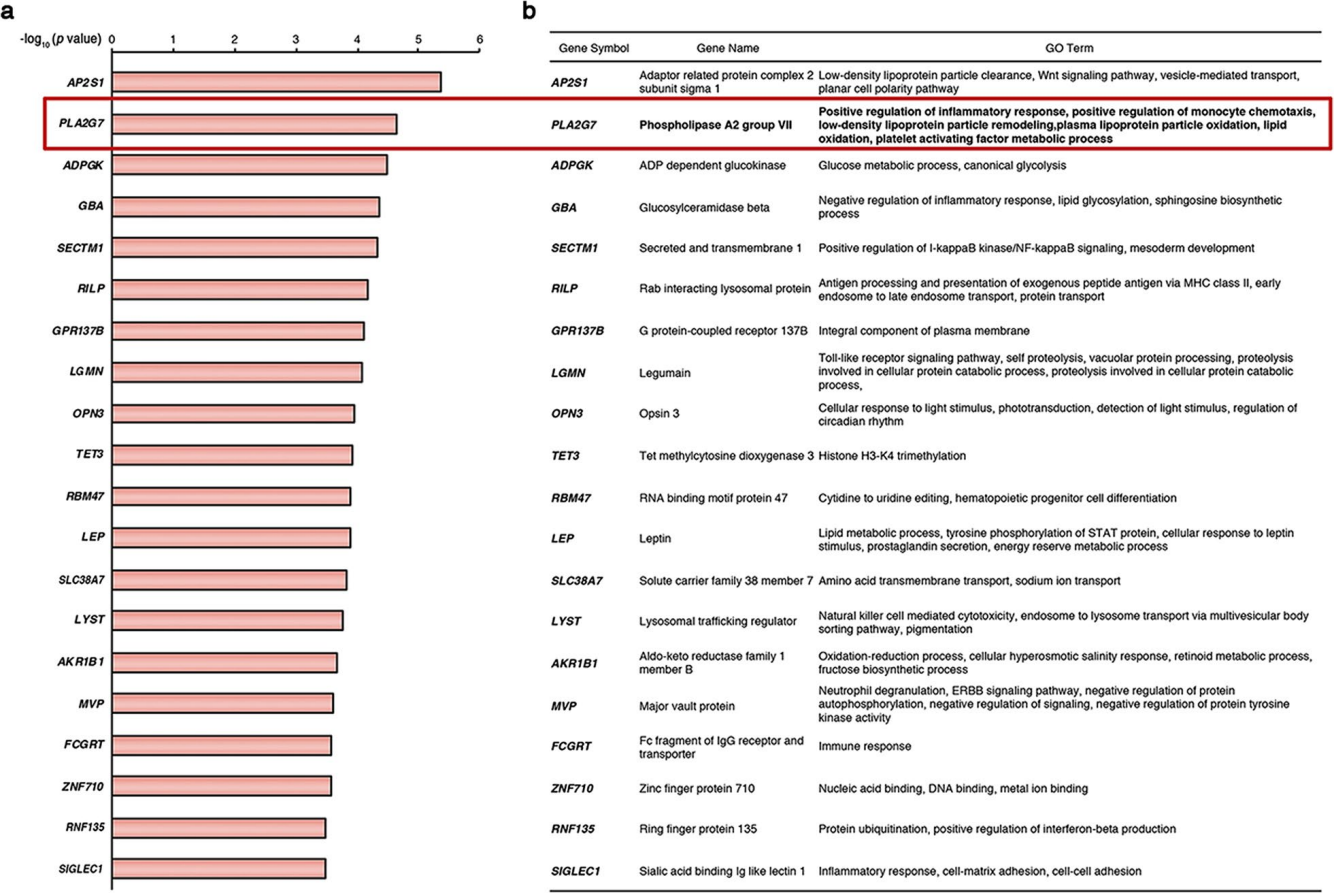
Top 20 vulnerability-specific hypomethylated genes with increased expression and their biological process. **a** The top 20 genes that were hypomethylated in SYM plaques and showing an increased expression in both advanced plaques and macrophages from ruptured plaques. These genes are presented in graph descending according to the log_10_ (*p* values). **b** Biological process GO terms of the top 20 genes. *PLA2G7* is highlighted with a red box. SYM, symptomatic; and GO, gene ontology

Among the 107 genes shown in Fig. 4a, *PLA2G7* stood out because the promoter of *PLA2G7* was one of the most significantly hypomethylated regions in SYM plaques, and its expression was upregulated in both advanced plaques and ruptured plaques (Fig. 5a, b). *PLA2G7* encodes Lp-PLA_2_, which is a key enzyme linking lipid homeostasis with inflammatory response [13]. *PLA2G7* related GO terms include positive regulation of inflammatory response, positive regulation of monocyte chemotaxis, low-density lipoprotein particle remodeling, lipid oxidation, and platelet-activating factor metabolic process. All these biological processes have been involved in the pathogenesis of plaque vulnerability.

**Fig. 6:**
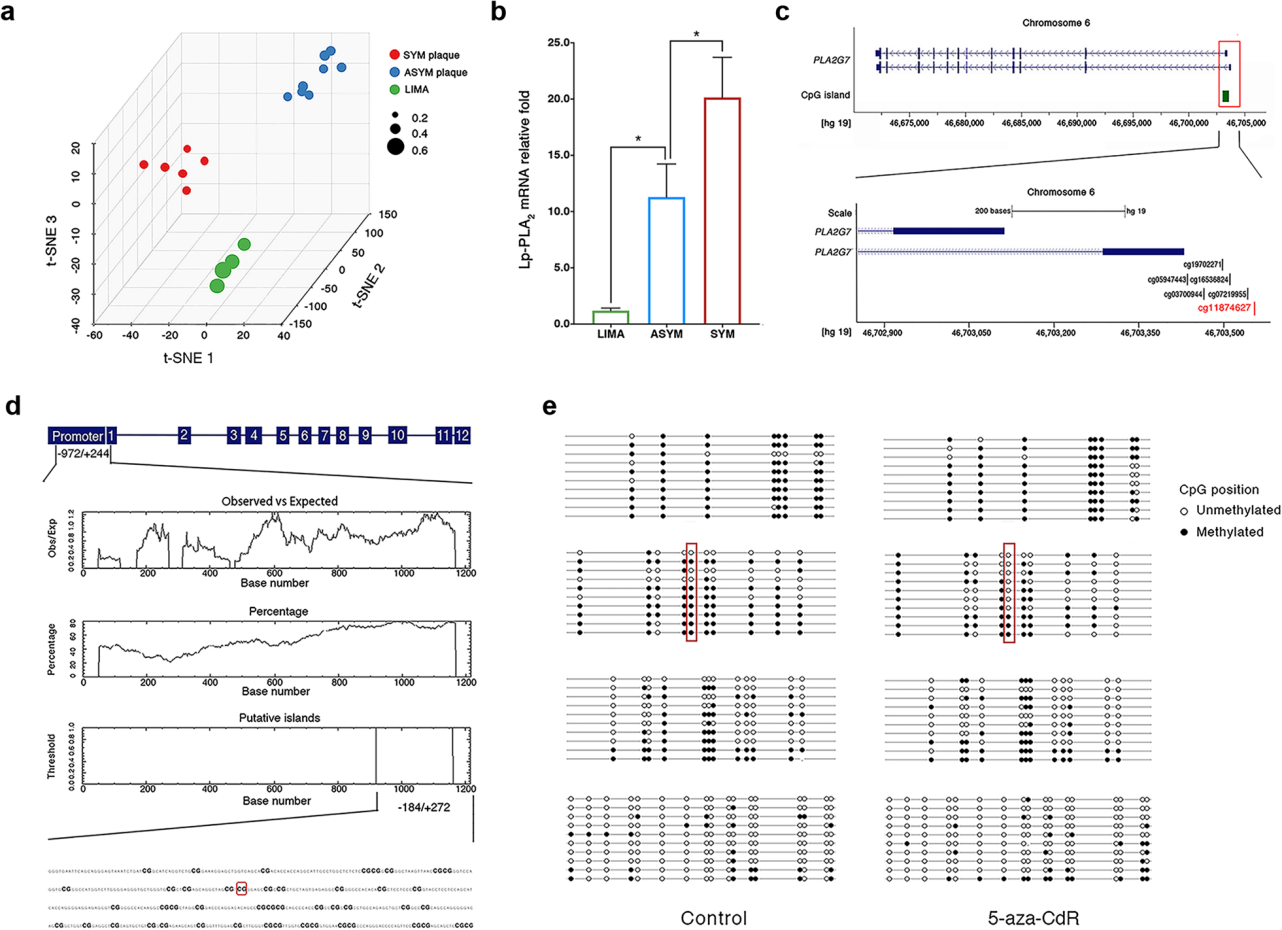
*PLA2G7*, a hypomethylated gene with increased expression in SYM and ASYM plaques. **a** t-SNE analysis diagram of *PLA2G7.* Red nodes represent SYM plaque samples (*n* = 6), blue nodes represent ASYM plaque samples (*n* = 8), and green nodes represent LIMA samples (*n* = 4). The node size represents the methylation level of *PLA2G7*. **b** The relative fold of the mRNA level of Lp-PLA_2_ in SYM plaques, ASYM plaques, and LIMAs (*n* = 6 for SYM plaques, *n* = 8 for ASYM plaques, and *n* = 4 for LIMAs, mean ± SD, ANOVA with a post hoc Bonfferoni test, *p < 0.05). **c** *PLA2G7* is composed of twelve exons (blue). The locations of the CpG islands in the promoter region relative to *PLA2G7* are shown according to the UCSC genome browser data (green). Six sites were found differentially methylated in the promoter region. Red highlights indicate one of the most significantly hypomethylated sites in plaques. **d** CpG plot of human *PLA2G7* gene illustrating the putative CpG islands in the promoter region. THP-1 cells was treated with LPS. Bisulfite sequencing of the promoter region was performed to detect the methylation status by DMSO (control) and methyltransferase inhibitor 5-aza-CdR (25 μM, 48 h, *n* = 3 different cell batches). The CpG dinucleotides are highlighted in black. The red box indicates significant methylation differences in site cg11874627. **e** DNA methylation of *PLA2G7* in THP-1 cells treated with LPS can be reversed by 5-aza-CdR. Bisulfite sequencing of the promoter region was performed to detect the methylation status by DMSO and methyltransferase inhibitor 5-aza-CdR (25 μM, 48 h, *n* = 3 different cell batches). A schematic diagram of the *PLA2G7* promoter is presented to display the CG dinucleotide methylation percentage and relative position. The node represents the status of methylation. The red box indicates significant methylation differences site cg11874627. SYM, symptomatic; ASYM, asymptomatic; LIMAs, human left internal mammary arteries; Lp-PLA_2_, lipoprotein-associated phospholipase A_2_; LPS, lipopolysaccharide; and TSA, trichostatin A

To determine the association between methylation changes of *PLA2G7* and plaque vulnerability, we used the t-SNE algorithm to classify the methylation states of *PLA2G7* in atherosclerotic plaques and lesion-free arterial vessel samples*.* All samples were distinctly separated into three groups based on the extent of hypomethylation: SYM plaques, ASYM plaques, and LIMAs, showing intra-group clustering but inter-group dissimilarity in the methylated states. Importantly, the hypomethylated level of *PLA2G7* significantly decreased with plaque destabilization and was the lowest in SYM plaques (Fig. 6a). Consequently, by using RT-qPCR, we found that the mRNA level of Lp-PLA_2_ was significantly (*p* < 0.05) upregulated in ASYM plaques compared with LIMA, and its expression was even higher in SYM plaques (Fig. 6b).

### A specific hypomethylated CpG site, cg11874627, was identified at the promoter region of *PLA2G7*

Next, we asked if the upregulation of *PLA2G7* expression is at least partially due to the hypomethylation of its promoter region in SYM plaques. There are six differentially methylated sites (cg11874627, cg03700944, cg16536824, cg19702271, cg0721995, cg05947443) in the promoter region of *PLA2G7 gene*, and cg11874627 was the most significantly hypomethylated site among them (Fig. 6c). We analyzed a genomic region ranging from − 972 to + 244 (1216 bp) around the transcription starting site of human *PLA2G7* gene. We found multiple CpG islands (defined as a genomic region of > 200 bp with a CG content of > 50% and an observed/expected CpG ratio of > 60%) in this promoter region (Fig. 5d). Interestingly, among the six differentially methylated sites, cg11874627 was found at one of these CpG islands (Fig. 6d).

We further delineated methylation status of the CpG sites at the promoter of the *PLA2G7* gene by bisulfite sequencing. This was performed in a human monocyte cell line, THP-1 cells, which was treated with lipopolysaccharide (LPS) to induce its differentiation into macrophages, as *PLA2G7* has been shown to be mainly expressed in monocytes derived macrophages [14]. We also treated the macrophages with 5-aza-2ʹ-deoxycytidine (5-aza-CdR), which pharmacologically inhibited the activity of DNA methyltransferases (DNMTs). Bisulfite sequencing revealed that methylation in the CpG islands, including cg11874627, was reduced after treatment with 5-aza-CdR, confirming that the *PLA2G7* is subjected to regulation by DNA methylation (Fig. 6e).

### Lp-PLA_2_ as a therapeutic target is regulated by DNA methylation via altering Sp3 binding

**Fig. 7:**
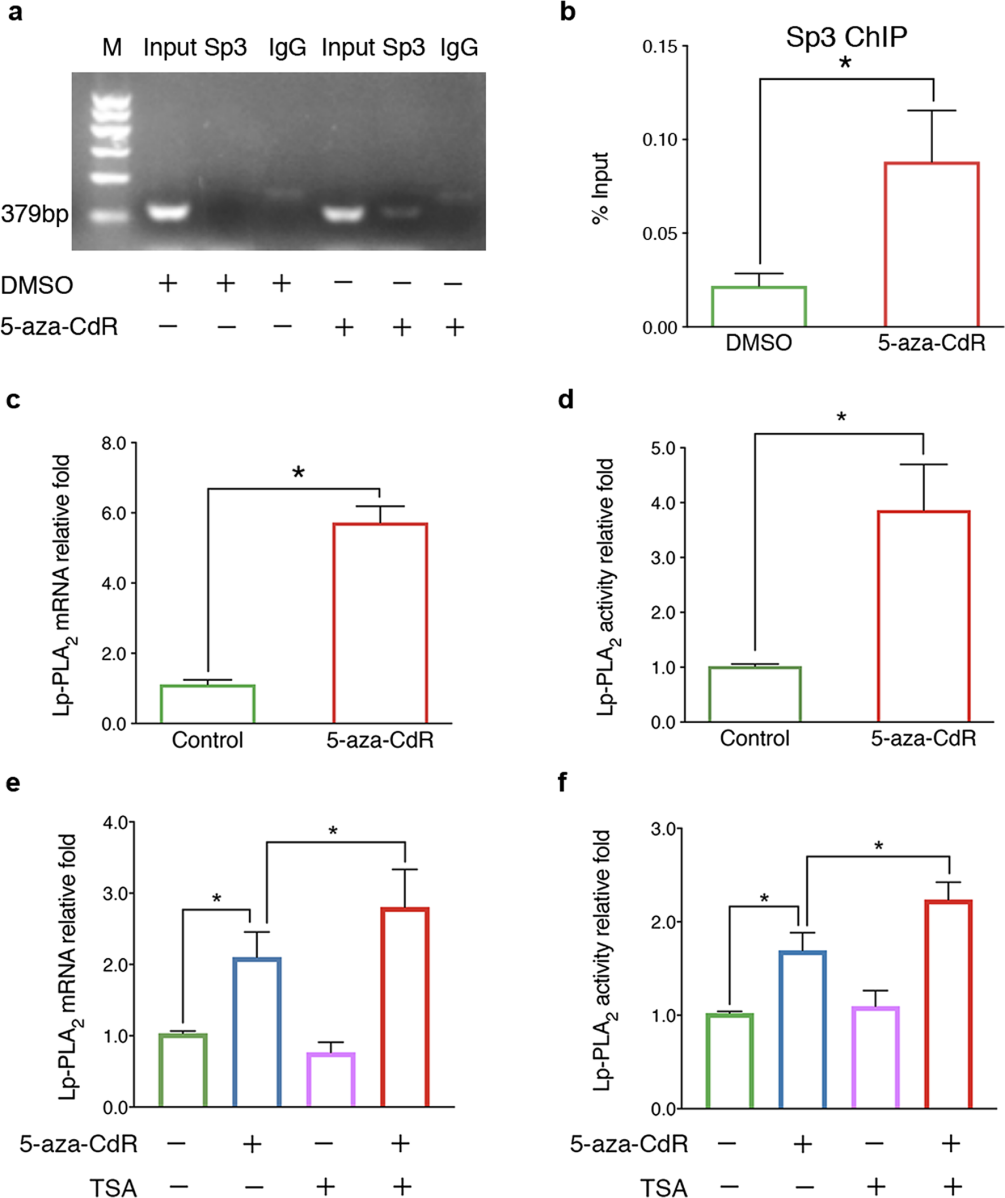
DNA methylation alters the binding of transcription factor Sp3 to the promoter of *PLA2G7* and changes gene expression. **a** ChIP assay on the promoter of *PLA2G7*. The effect of 5-aza-CdR on Sp3 binds to the 5ʹ-flanking regions of *PLA2G7*. THP-1 cells were treated with 5-aza-CdR (25 μM, 48 h). ChIP assays were performed with the antibodies against Sp3 and IgG were used as a negative control. Three independent experiments were done with the similar results. Results from one experiment are shown. **b** qPCR of ChIP assay in (**a**). Precipitated DNA fragments were detected using qPCR with specific primers spanning the DNA segments containing the indicated Sp3 responsive element of *PLA2G7* promoter. Three independent experiments were done with similar results, each with triple biological repeats. Data were from one experiment with three technical replicates. *n* = 3 different cell batches, means ± SD, Student’s *t*-test, *p < 0.05. **c** The level of Lp-PLA_2_ mRNA in THP-1 cell pretreated with LPS with or without (control) 5-aza-CdR (25 μM, 48 h). **d** The level of Lp-PLA_2_ activity in THP-1 cell pretreated with LPS with or without (control) 5-aza-CdR (25 μM, 48 h). For **c** and **d** *n* = 3 different cell batches, mean ± SME, Mann–Whitney U test, *p < 0.05. **e** The level of Lp-PLA_2_ mRNA in THP-1 cell pretreated with LPS in the presence (+) or absence (−) of 5-aza-CdR (25 μM, 48 h) and TSA (100 nM, 24 h). **f** The level of Lp-PLA_2_ activity in THP-1 cell pretreated with LPS in the presence (+) or absence (−) of 5-aza-CdR (25 μM, 48 h) and TSA (100 nM, 24 h). For **e** and **f** *n* = 3 different cell batches, mean ± SME, ANOVA with a post hoc Bonfferoni test. *p < 0.05. 5-aza-CdR, 5-aza-2'-deoxycytidine; Lp-PLA_2_, lipoprotein-associated phospholipase A_2_; ChIP, chromatin immunoprecipitation; LPS, lipopolysaccharide; and TSA, trichostatin A

Sp3 has been previously identified as a transcription factor binding to the promoter region of *PLA2G7* and regulates its expression [15]. Interestingly, the hypomethylated CpG site cg11874627 is also located at a Sp3 binding motif. To determine whether the methylation status of this site affects the binding of Sp3, we performed chromatin immunoprecipitation (ChIP) assay using Sp3 antibody. We found that the demethylated agent 5-aza-CdR increased the level of DNA harboring Sp3 binding sites co-purified with Sp3, demonstrating that demethylation of the region enhanced the binding of Sp3 (Fig. 7a, b). In line with this, we showed that 5-aza-CdR treatment promoted Lp-PLA_2_ expression at both mRNA and protein levels in a dose-dependent manner (Fig. 7c, d, Additional file 9). Treating cells with trichostatin A (TSA), an inhibitor of histone deacetylase, leads to a more open chromatin configuration and a general increase in DNA accessibility for transcription factor binding to facilitate demethylation [16]. We found that both levels of Lp-PLA_2_ activity and mRNA were enhanced by the co-treatment with 5-aza-CdR and TSA, but not by the treatment of TSA alone (Fig. 7e, f). Thus, our data suggested that hypomethylation of the promoter region of the *PLA2G7* gene facilitated the binding of transcription factor Sp3, thereby increasing Lp-PLA_2_ expression in SYM atherosclerotic plaques, and the latter was further enhanced by deacetylase.

## Discussion

No genome-wide association study has been conducted to investigate the role of DNA methylation in the vulnerability of atherosclerotic plaques so far. In the present study, we have identified global alterations in DNA methylation in SYM and ASYM atherosclerotic plaques and characterized specific features of plaque vulnerability by identifying a total of 90,368 notable discrepant methylated sites corresponding to 14,657 annotated genes (FDR < 0.05, |delta beta|> 0.03). Notably, we found that among the differential methylated genomic sites, 30% were located at the promoter regions, 14% were in the CpG islands, and 67% of these displayed hypomethylation. Subsequently, we focus on CpG islands which located at promoter regions of protein-coding genes and revealed 14,596 differentially methylated sites (|delta beta|> 0.1) located at the promoter regions of 7048 genes, which included 9863 hypomethylated (corresponding to 4695 genes) and 4733 hypermethylated (corresponding to 2353 genes) sites. Further GO and pathway analysis revealed that the biological processes of these genes with differential methylation were enriched in various aspects of the immune response and inflammation. This strongly suggested that inflammation and immune activity were subjected to epigenetic regulation and were involved in driving lesion progression and plaque destabilization. Moreover, we uncovered the epigenetic regulation mechanism of *PLA2G7*, a gene critical in regulating lesion stability, was one of the top hypomethylated genes with increased expression in SYM plaques. We identified a specific binding motif of transcription factor Sp3 (cg11874627) in the hypomethylated CpG site in the promoter region of *PLA2G7*. Demethylation of this region promoted the binding of Sp3 and increased the expression of *PLA2G7*.

Genome-wide studies offer a comprehensive strategy to elucidate mechanisms of gene regulation in cardiovascular disease, such as genome-wide association studies (GWAS) focusing on genetic variation among individuals [17]. However, these studies drew only part of the heritable component of the diseases [9]. Emerging evidence has suggested the importance of epigenetic modifications, particularly DNA methylation, as a new layer of biological regulation in cardiovascular disease [18, 19]. Several vascular cells, including endothelial cells, vascular smooth muscle cells, and monocytes/macrophages in atherosclerosis harbor epigenetic alternations, which complement genetic abnormalities [20, 21]. Nevertheless, the evidence showing the DNA methylation regulation in driving lesion progression and contributing to plaque vulnerability remains largely unresolved. Since the vulnerability of atherosclerotic lesion is a crucial feature that determines the clinical sequelae, the underlying epigenetic alterations need to be further elucidated. Here, we characterized the DNA methylome signature of plaque vulnerability. We revealed that DNA hypomethylation sites were twofold more numerous than hypermethylated sites in SYM plaques, suggesting that the hypomethylated modification was predominant in vulnerable plaques.

The integrated analysis of DNA methylation modifications and gene expression profiles provides an important basis for understanding the role of epigenetic mechanisms in the pathophysiology of the vulnerability of atherosclerosis. We found that the function of genes with differentially methylated promoters in vulnerable plaques was mainly leukocyte activation/differentiation, cell migration/adhesion, immune system process, B cell/lymphocyte-mediated immunity. Our findings support previous studies which indicated that vascular inflammation is a key component of atherosclerosis that contributes to plaque instability and clinical cardiovascular events, including ischemic MI and stroke [22, 23].

Recent studies assessed the methylation status of some individual genes and indicated that the expression levels of inflammatory genes such as *AIRE1*, *ALOX12*, and *IL-6* appear to be regulated through DNA methylation [8, 9, 24]. Changes in the methylation status of these “target” genes may affect the functional pathways involved in atherosclerotic plaque progression and vulnerability. Subsequently, we identified epigenetic changes of pro- and anti-inflammatory mediators in SYM plaques compared with ASYM plaques and revealed that a variety of cytokines and chemokines were subjected to DNA methylations. Our results demonstrated that the plaque destabilization was a complex epigenetic regulatory process, in which the “tug of war” between pro-inflammatory and anti-inflammatory mediators in the atherosclerotic lesion directly contribute to the vulnerable plaque and clinical consequences. Additionally, DNA methylation shows substantial variation across tissue types as well as individual cell types. The total effect of a phenotype on DNA methylation can be decomposed into two components, one representing the effect of phenotype on proportions of major cell types, the other representing the differences and global effects at focused loci. Due to different compositions of the cell type between the vulnerable and non-vulnerable plaques, besides the global effects at focused loci on DNA methylation, the proportions of major cell types, especially the inflammatory cells (mainly macrophages), may also have effect on DNA methylation. It was not adequately separate cell composition methylation effects from those disease-associated DNA methylations in the present study. Further studies in specific cell populations are warranted to precisely assess the pathobiological of plaque destabilization and progression.

Integrated analysis of methylation and gene expression profiles identified 107 genes with hypomethylation and upregulated expression. The most pronounced gene is *PLA2G7* in differential hypomethylation, which encodes Lp-PLA_2_ [13]. Lp-PLA_2_ is mainly secreted by macrophages and rapidly degrades oxidatively modified phospholipids in low-density lipoproteincholesterol (LDL-C), leading to the formation of two potent pro-inflammatory factors, lysophosphatidylcholine (lysoPC), and oxidized non-esterified fatty acids (oxNEFAs) [13]. Several extensive cohort studies showed that individuals with elevated Lp-PLA_2_ level and/or activity are associated with an increased risk of cardiovascular death, MI, and stroke [25, 26]. Moreover, an increased level of Lp-PLA_2_ was found in cap fibroatheromas and ruptured plaques but is almost absent in stable lesions in human [27]. Animal experiments in hypercholesterolemic diabetic swine showed that direct inhibition of Lp-PLA_2_ activity by selective inhibitor darapladib could stave off the progression to advanced atherosclerotic lesions by reducing the plaque area and the mean necrotic core area [28].

Expression of Lp-PLA_2_ is regulated through various mechanisms on multiple interconnected levels, including transcriptional, post-transcriptional, and translational levels [29]. Besides regulatory mechanisms revealed by previous studies, epigenetic mechanisms, in particular DNA methylation, are now gaining considerable attention, as they provide another vital layer in gene regulation. To the best of our knowledge, this is the first report to demonstrate that Lp-PLA_2_ expression was regulated by DNA methylation via altering the transcription factor Sp3 binding to the promoter region of *PLA2G7*. Sp3 participates in the macrophage-specific expression of many genes, which play essential roles in the maturation of monocytes to macrophages [15]. Our results show that demethylation of the promoter region of the *PLA2G7* gene using 5-aza-CdR facilitated the binding of transcription factor Sp3. These indicated that hypomethylation modulates the chromatin to be an open conformation accessible to the transcription factor Sp3 binding, which further drives Lp-PLA_2_ expression. Notably, the expression of Lp-PLA_2_ was further upregulated by deacetylation, suggesting that in combination with hypomethylation, deacetylation may reduce the affinity of histone for DNA resulting in the formation of a more relaxed chromatin structure, further increasing chromatin accessibility and Lp-PLA_2_ expression. Our findings were consistent with a recent study showing that inhibition of DNMTs was associated with gene expression alteration and was enhanced by histone deacetylase inhibitors [30].

Both basic and clinical studies support the causality of the potent pro-inflammation mediator Lp-PLA_2_ to plaque vulnerability; however, successful translation of therapeutic intervention of Lp-PLA_2_ into clinical benefits has proven to be challenging. In SOLID-TIMI 52 (the stabilization of plaque using darapladib-thrombolysis in myocardial infarction 52) trial [31], direct inhibition of Lp-PLA_2_ with darapladib in patients with ACS failed to reduce the risk of future cardiovascular death, MI, or urgent coronary revascularization for myocardial ischemia. Some possible explanations for failed efficacy of Lp-PLA_2_ inhibitors have been overlooked. Compared with concurrent therapy with statin that diminish the risk of cardiovascular events, the rationality of the inclusion criteria for the population under study is perhaps crucial. Lp-PLA_2_ is critical in determining plaque instability via inflammatory pathways. Consequently, it would be better if the SOLID-TIMI 52 trial screened and classified the high sensitivity C-reactive protein (hsCRP) level of patients to evaluate the efficacy of darapladib in patients with a pro-inflammatory constitution. Similarly, in CIRT (Cardiovascular Inflammation Reduction Trial) [32], treatment with inflammation inhibition methotrexate did not result in fewer cardiovascular events among patients in a median hsCRP level of only 1.5 mg/L at baseline. On the contrary, CANTOS (Canakinumab Anti-inflammatory Thrombosis Outcomes Study) demonstrated that IL-1β inhibitor canakinumab significantly reduced cardiovascular events in post-myocardial infarction patients with hsCRP levels ≥ 2 mg/L [33]. Recently, COLCOT (Colchicine Cardiovascular Outcomes Trial) showed colchicine led to a substantially lower risk of ischemic cardiovascular events in patients with a pro-inflammatory constitution [34]. These findings renewed the interest in therapeutic agents that target inflammatory pathways in cardiovascular disease, including colchicine (LoDoCo2, ACRTN12614000093684, CLEAR-SYNERGY, NCT03048825 and CONVINCE, NCT02898610), IL-2 (LILACS, NCT03113773), and agents that act on the IL-1β/IL-6 signaling pathway. Thus, the poor patient specificity per se, rather than the ineffective Lp-PLA_2_ inhibitor, at least partially accounted for the negative results of the SOLID-TIMI 52 trial. More precise studies to reassess the potential benefits of targeting Lp-PLA_2_ in patients with sufficient inflammatory burden at the base level are warranted. Although the results of these trials will be critical in framing the future role of targeting inflammation, it is notable that IL-1β inhibition and colchicine only moderately (~ 15% and ~ 23%, respectively) reduced cardiovascular events, leaving a high residual risk. Furthermore, the failure of the CIRT also showed that broad anti-inflammatory treatments are ineffective in reducing cardiovascular events. Altogether these observations underscore that anti-inflammatory treatments must be tailored to specific targets rather than broad-spectrum inflammatory response towards atherosclerosis. Thus, Lp-PLA_2_ is a promising target and represents an opportunity to treat individuals at risk selectively.

More importantly, we identified the expression of Lp-PLA_2_ was modulated by methylation during plaques progression, which will help to design novel therapeutic strategies targeting the epigenetic regulation. Epigenetic processes are highly reversible, dynamic, and hence drug targetable, providing an excellent opportunity to treat atherosclerosis [35]. Although the findings of the clinical trials do not support a direct therapeutic role for Lp-PLA_2_, targeting the DNA methylation of Lp-PLA_2_ might offer a potential therapeutic strategy to hinder the transition from vulnerable to stable plaque. A vulnerable plaque state may influence causally both the epigenetics in the upstream and the true clinical efficacy endpoint rather than the inflammatory mediators actually lying in the down-stream pathway from disease to clinical events. Notably, several epigenetic drugs have already been approved for the treatment of several conditions, including cancer [36]. Of note, pharmacological inhibition of DNMTs by 5-aza and its analogs inhibit experimental atherosclerosis induced either by atherogenic diets or partial ligation surgery [37]. More recently, two landmark studies have independently demonstrated the important role of the hematopoietic DNA demethylating enzyme TET2 in preventing atherosclerosis by repressing pro-inflammatory cytokine and chemokine expression as well as inflammasome activation [38, 39]. In the present study, hypomethylation of *PLA2G7* in vulnerable plaque suggests the necessity to develop epigenetic drugs targeting DNA methylation states of *PLA2G7* to combat plaques destabilization, which will be a challenging yet worthwhile path. For example, it is possible to methylate cg11874627 to reduce PLA2G7 expression with CRISPR technology [40].

The present study has several limitations. First, the methylome was profiled by using the array technology that does not cover all the possible methylation sites. Moreover, methylation probes that overlap with genetic polymorphisms have been removed during the analysis. Thus, some differential methylated sites may be missed. To this end, whole-genome bisulfite sequencing could be alternative technology with broader coverage but at a higher cost. Secondly, the number of samples included in this study is relatively small. However, we detected two differential phenotypes of plaques, the vulnerable and non-vulnerable plaques, which was more important to minimize the discrepancies due to the heterogeneity of cell types in the atherosclerotic plaques and lesion free vessels that were used in the previous studies [41, 42]. Finally, our ability to use the DNA methylome data to identify novel pathways and relationships was limited because our functional enrichment and network analyses were based on known biological processes and gene-product interactions.

## Conclusions

Genome-wide DNA methylation analyses of vulnerable and non-vulnerable atherosclerotic lesions highlight the extensive modifications in DNA methylation, especially hypomethylation in plaque destabilization. Differential methylation affected a high percentage of genes involved in inflammatory and immune response, which contribute to the pathogenesis of plaques destabilization. Importantly, the hypomethylation of *PLA2G7* gene upregulates its expression by increasing the binding of transcription factor Sp3. Our findings extend the epigenetic remodeling in plaque destabilization and offer a promising novel epigenetic therapeutic target for atherosclerosis.

## Materials and methods

### Human specimens and clinical characteristics of the patients

Patients with extracranial high-grade internal carotid artery stenosis undergoing CEAs in China-Japan Friendship Hospital were enrolled (*n* = 14) in the study. SYM patients were defined as having had an acute cardiovascular event (stroke, transient ischemic attack) within six months according to validated criteria [43]. ASYM patients had no acute cardiovascular events and any other relevant symptoms within six months before elective surgery [44]. The atherosclerotic plaques were obtained from the SYM and ASYM patients. Additionally, human LIMA tissue samples were collected from patients undergoing coronary artery bypass surgery (CABG) procedure (*n* = 4). The samples were immediately snap-frozen and kept in liquid nitrogen until DNA methylation and histology analysis. HE staining of LIMAs and atherosclerotic plaques were showed in Additional file 10. All procedures involving humans were approved by the Institutional Ethics Review Board of China-Japan Friendship Hospital (No. 2019-85-K56) and conformed to the standards of the Declaration of Helsinki. All participants provided written informed consent before enrollment in this study.

### DNA and RNA extraction

DNA was extracted from frozen tissue specimens using the QIAamp DNA Mini Kit (Qiagen), and RNA was extracted from cell lines using the RNAsimple Kit (Tiangen) following the manufacturer’s instructions. Qualities of the DNA and RNA samples were analyzed using Multiskan FC Photometer (Thermo Fisher Scientific).

### DNA methylation profiling

All DNA samples were subjected to bisulfite modification using the EZ DNA Methylation Kit (Zymo Research) according to the manufacturer’s instructions. Briefly, 1 μg genomic DNA was subjected to bisulfite conversion and kept in 20 μl elution buffer. Then, 5 μl of each sample was profiled using Human Infinium Methylation 450Kor MethylationEPIC BeadChip (Illumina). The details for distribution of sample types between Human Infinium Methylation 450 K and MethylationEPIC BeadChip are shown in Additional file 11. With these chip arrays, methylation was quantitatively measured at 450,000 or 850,000 CpG loci. These loci covered 99% of the RefSeq genes and were distributed across whole gene, including promoters, gene bodies, and 3ʹ-UTRs [45].

### Bioinformatics analysis

Array data were evaluated and preprocessed using the minfi R package (version 1.32.0) [46]. The 450 K and EPIC data were merged with the combineArrays function. The batch effect was corrected with the sva R package (version3.34.0) [47]. Differential methylation was determined using the IMA R package (version 3.1.2) [45]. The CpG loci on the X and Y chromosomes were excluded from the analysis to eliminate any gender bias. We used the *t*-test to determine statistical significance. FDR was used for multiple testing correction, and FDR < 0.05 was considered statistically significant. |delta beta| was set at > 0.03 or > 0.1.

Circos (version 0.67) was used to visualize the methylation pattern of the whole genomic landscape of different groups [48]. GO analysis was performed using ClueGO (version 2.5.4) and DAVID (https://david.ncifcrf.gov/).

Integration analysis with expression data was performed as follows: the DNA microarray data from early vs. advanced carotid plaque (GSE28829) and the microarray data of macrophages from human ruptured vs. stable plaque (GSE41571) were downloaded from the Gene Expression Omnibus (GEO) database (http://www.ncbi.nlm.nih.gov/geo/) [11, 12]. The raw array data were analyzed using SAM, and genes with FDR < 0.05 were considered to be differentially expressed [49]. Venn diagrams were made using the VennDiagram R package (version1.6.20) to compare gene lists of hypomethylation and increased expression in atherosclerotic plaques [50].

Power estimated by a two-side two-sample unequal-variance t-test. The methylated value of CpG site of *PLA2G7* was used for power calculation. The group sample size of SYM (*n* = 6) and ASYM plaques (*n* = 8) achieved 92.989% power to reject the null hypothesis of equal means. The *PLA2G7* gene was analyzed as follows: The samples were clustered with the Rtsne R package (version 0.15) according to the beta values. Genomic loci were visualized with the UCSC genome browser [51]. The CpG islands upstream 1200 bp (− 972/+ 244) were visualized using CpG Plot (http://www.ebi.ac.uk/Tools/seqstats/emboss_cpgplot). Transcription factor binding sites in the promoter of *PLA2G7* were identified by using Jaspar [52].

### Cell culture and treatments

THP-1 cells were obtained from Cell Resource Center, Institute of Basic Medical Sciences, Chinese Academy of Medical Sciences, and cultured in RPMI-1640 medium with 10% FBS (Gibco) at 37 °C in a humidified incubator with 5% CO_2_. To probe the transcription factor binding THP1 cells were treated with 25 μM of 5-aza-CdR (Sigma) for 48 h and then 100 nM TSA (Sigma) for 12 h. All experiments were three biological replicates.

### Bisulfite sequence

Genomic DNA was extracted from THP-1 cells with the QIAamp DNA Mini kit (Qiagen). DNA was treated with sodium bisulfite as previously described [53]. PCR and sequencing primers (Additional file 12) were designed using Primer5. The PCR was performed using the following program: 95 °C for 15 min, followed by 40 cycles of 95 °C for 60 s, 50 °C for 30 s and 72 °C for 30 s, and ended with incubation at 60 °C for 10 min. The PCR products were analyzed by electrophoresis in 1.0% agarose gels. The PCR products were TA cloned, and ten clones of each amplified fragment were sequenced using ABI automated DNA sequencer (Applied Biosystems).

### Chromatin immunoprecipitation (ChIP)

ChIP assays were performed with a ChIP kit (Cell Signaling Technology) according to the manufacturer's recommendations. Briefly, three biological replicates of THP-1 cells were treated with control (DMSO) or 5-aza-CdR for 48 h. For ChIP assays each assay 12 × 10^6^ cells were cross-linked with 1% formaldehyde with protease inhibitor at room temperature for 10 min. Chromatin was fragmented to size of 150-900 bp by sonication(Covaris 220). Immunoprecipitation was performed by adding 2 μg anti-Sp3 antibody (sc-28305, Santa Cruz Biotechnology) or unspecific rabbit IgG (Santa Cruz Biotechnology) overnight at 4 °C under rotation. Each immunoprecipitated sample was added to protein G agarose beads for 3 h of incubation at 4 °C. After pull-down, beads were washed five times. The immunocomplexes were extracted and processed by reverse cross-linking, proteinase K digestion, and DNA purification. qPCR was performed to amplify the precipitated DNA with the primers of *PLA2G7* (5'-TCACAGTGCCAACTGAGAGA-3'; 5'-CAAGTTGGTCTCCAGGGCAT-3') flanking the Sp3 responsive elements (5ʹ-AGGCTAGCGTCGGGAGCCGC-3').

### Real-time quantitative polymerase chain reaction (RT-qPCR)

RT-qPCR was performed as described previously [54]. Briefly, total RNA was reverse transcribed to cDNA with M-MLV reverse transcriptase with random primers (Promega). RT-qPCR was performed with SYBR-green dye and Taq polymerase using the 7Dx real-time system (Applied Biosystems). Gene expression was quantified using the comparative CT method, normalized to GAPDH, and expressed as fold induction of control. Primer sequences are presented in Additional file 12.

### Enzyme-linked immunosorbent assay (ELISA)

The protein level of Lp-PLA_2_ was measured using the ELISA kit (USCN Life Science) according to the manufacturer’s instructions.

### Statistical analysis

The normality of data distribution was tested using the Wilk–Shapiro normality test. Normally distributed data, presented as mean ± standard deviation (SD), were compared by unpaired Student’s *t*-test for two groups comparisons and one-way analysis of variance (ANOVA) and followed up with a post hoc Bonfferoni test for multiple comparisons. Categorical variables, presented as *n* (%), were analyzed by the chi-square test or Fisher’s exact test, as appropriate. All *p* < 0.05 from two-sided tests were accepted as statistically significant. All statistical analyses were performed using SPSS 23.0 software (SPSS Inc., Chicago, Illinois) and GraphPad Prism 7 (GraphPad Software, La Jolla, California).

## Supplementary Information

**Additional file 1.** Schematic workflow of sample origin and the techniques used in atherosclerotic plaques and LIMAs

**Additional file 2.** Differentially methylated sites of vulnerable and non-vulnerable plaques

**Additional file 3.** The epigenome-wide signature of DNA methylome in atherosclerotic plaques and LIMAs.

**Additional file 4.** GO analysis of the genes differentially hypomethylated in SYM and ASYM plaques

**Additional file 5.** GO analysis of the genes differentially hypermethylated in SYM and ASYM plaques

**Additional file 6.** The biological function of the genes differentially methylated in atherosclerotic plaques and LIMAs

**Additional file 7.** Genes list of hypomethylated in SYM vs. ASYM plaques and upregulated in rupture vs. stable (GSE41571) and early vs. advanced (GSE28829) plaques

**Additional file 8.** GO analysis of the genes hypomethylated in SYM vs. ASYM plaques and upregulated in rupture vs. stable (GSE41571) and early vs. advanced (GSE28829) plaques

**Additional file 9.** Expression of Lp-PLA2 mRNA treated with different concentration of 5-aza-CdR

**Additional file 10.** HE staining of human left internal mammary arteries and atherosclerotic plaques

**Additional file 11.** Distribution of SYM plaques, ASYM plaques, and LIMAs between Human Infinium Methylation 450K and MethylationEPIC BeadChip

**Additional file 12.** Sequences for primers

## Data Availability

Data supporting the findings of this study are available within the manuscript and supplementary information and are also available from the authors upon reasonable request. The raw BeadChip data are available in the NCBI GEO under accession number GSE149759, https://www.ncbi.nlm.nih.gov/geo/query/acc.cgi?acc=GSE149759.

## Abbreviations

ASCVD: atherosclerotic cardiovascular disease
ASYM: asymptomatic
CABG: coronary artery bypass graft
CEAs: carotid endarterectomies
ChIP: chromatin immunoprecipitation
DAVID: database for annotation, visualization, and integrated discovery
DNMTs: DNA methyltransferases
GEO: gene expression omnibus
GO: gene ontology
GWAS: genome-wide association studies
hsCRP: high sensitivity C-reactive protein
IL: interleukin
LDL-C: low-density lipoprotein cholesterol
LIMA: left internal mammary artery
Lp-PLA_2_: lipoprotein-associated phospholipase A_2_
LPS: lipopolysaccharide
lysoPC: lysophosphatidylcholine
MI: myocardial infarction
oxNEFAs: oxidized nonesterified fatty acids
SYM: symptomatic
TNF: tumor necrosis factor
TSA: trichostatin A
UTRs: untranslated regions
5-aza-CdR: 5-aza-2'-deoxycytidine
