# Supplementary material for: DNA methylome profiling reveals epigenetic regulation of lipoprotein-associated phospholipase A2 in human vulnerable atherosclerotic plaque
Source: Clin Epigenetics. 2021 Aug 21;13:161. doi: 10.1186/s13148-021-01152-z (PMC8379831; doi:10.1186/s13148-021-01152-z)
Supplement: Supplementary file 1 — Additional file 1. Schematic workflow of sample origin and the techniques used in atherosclerotic plaques and LIMAs [file 13148_2021_1152_MOESM1_ESM.pdf]

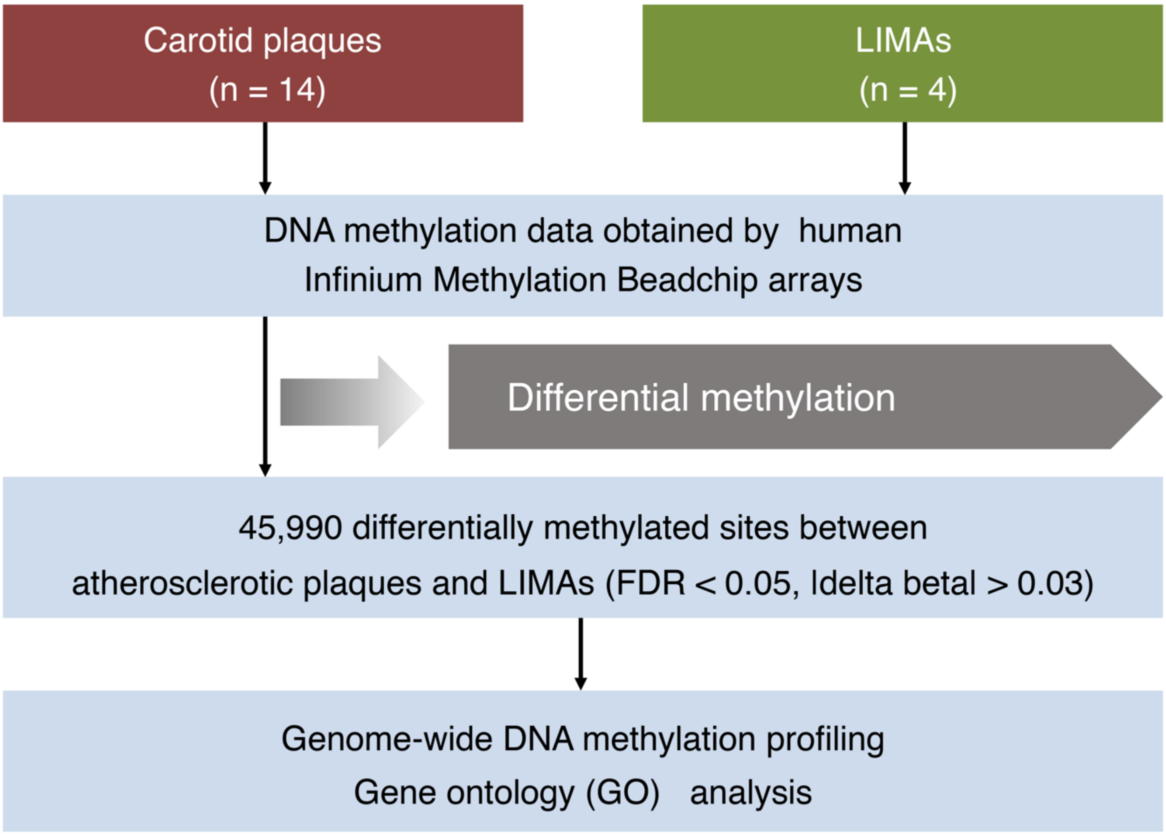

Additional file 1. Schematic workflow of sample origin and the techniques used in atherosclerotic plaques and LIMAs. LIMAs, left internal mammary arteries.
