# Supplementary material for: DNA methylome profiling reveals epigenetic regulation of lipoprotein-associated phospholipase A2 in human vulnerable atherosclerotic plaque
Source: Clin Epigenetics. 2021 Aug 21;13:161. doi: 10.1186/s13148-021-01152-z (PMC8379831; doi:10.1186/s13148-021-01152-z)
Supplement: Supplementary file 3 — Additional file 3. The epigenome-wide signature of DNA methylome in atherosclerotic plaques and LIMAs. [file 13148_2021_1152_MOESM3_ESM.pdf]

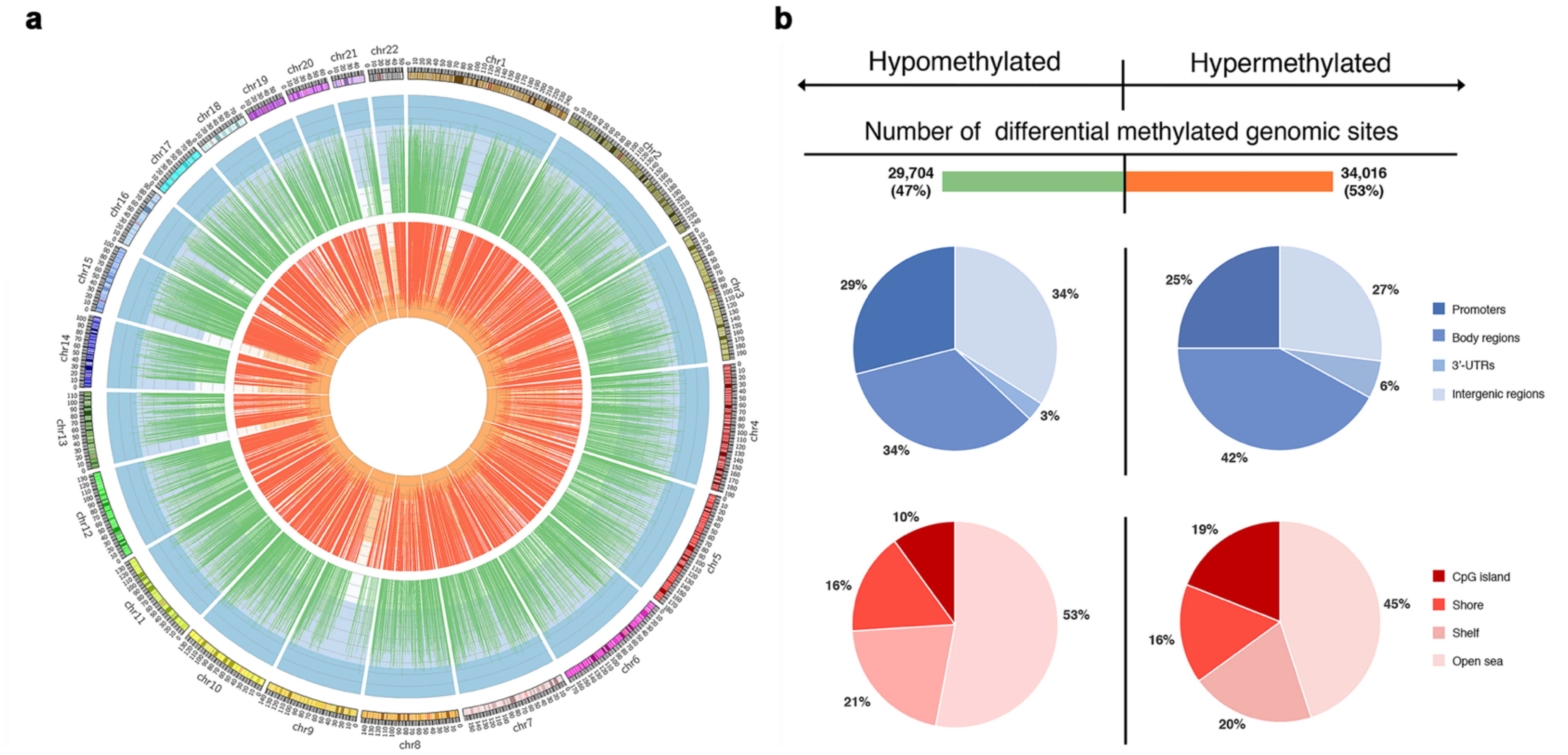

Additional file 3. The epigenome-wide signature of DNA methylome in atherosclerotic plaques and LIMAs. (**a**) Circos graph of DNA methylation levels for atherosclerotic plaques compare with LIMAs. The outer circle displays human chromosomes with scale at 1MB bins. The hypo-methylation probes are displayed in green lines and hyper-methylation probes are displayed in red lines. The height of blue background indicates scale of beta-score in DNA methylation assay. (**b**) Hypo- and hyper-methylated in atherosclerotic plaques compared with LIMAs are displayed on the left and right panels, respectively. LIMAs, left internal mammary arteries; UTRs, untranslated regions.
