# Supplementary material for: DNA methylome profiling reveals epigenetic regulation of lipoprotein-associated phospholipase A2 in human vulnerable atherosclerotic plaque
Source: Clin Epigenetics. 2021 Aug 21;13:161. doi: 10.1186/s13148-021-01152-z (PMC8379831; doi:10.1186/s13148-021-01152-z)
Supplement: Supplementary file 6 — Additional file 6. The biological function of the genes differentially methylated in atherosclerotic plaques and LIMAs [file 13148_2021_1152_MOESM6_ESM.pdf]

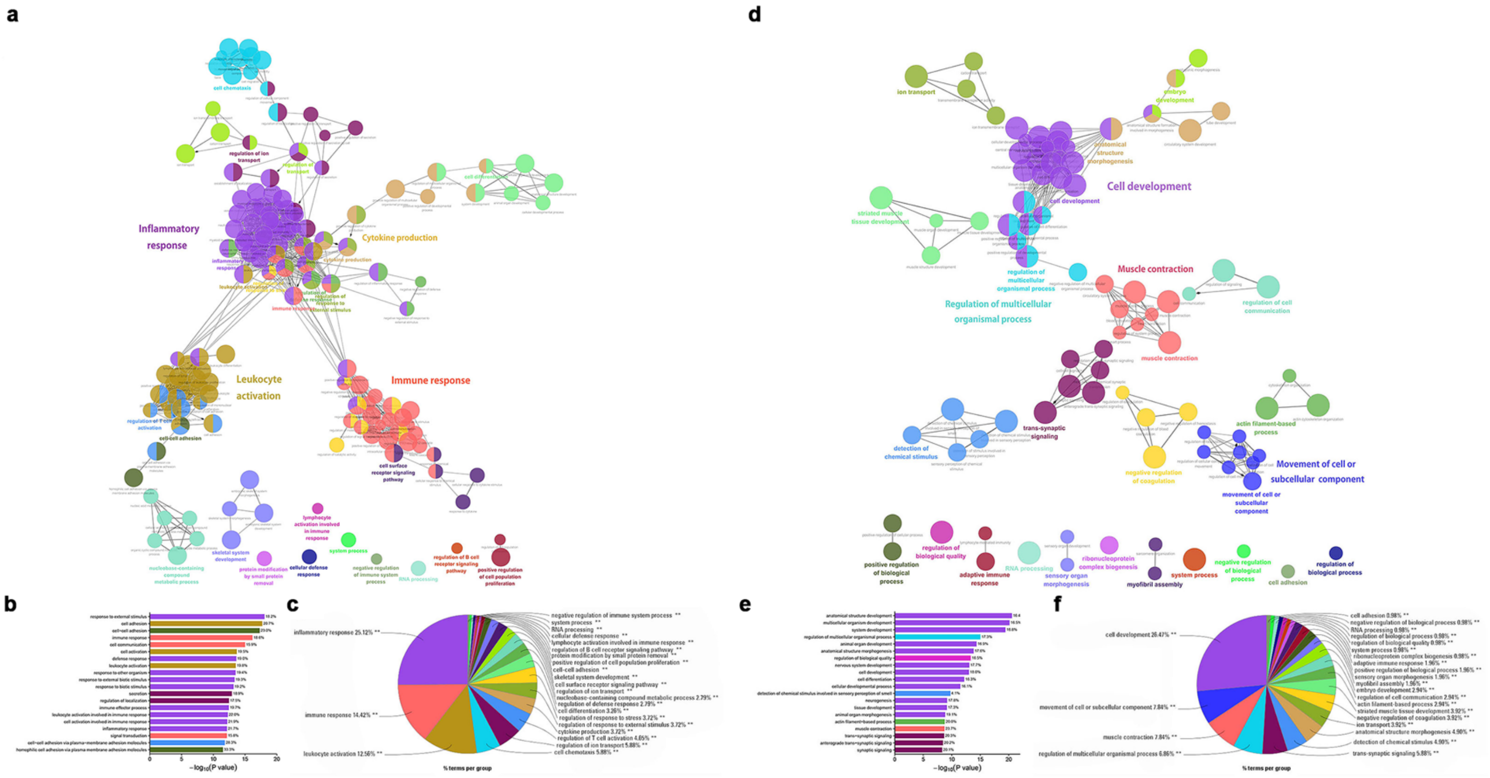


**Additional file 6. The biological function of the genes differentially methylated in atherosclerotic plaques and LIMAs.** (**a, d**) GO analysis of hypomethylated and hypermethylated genes in atherosclerotic plaques compared with LIMAs using ClueGO and DAVID. Nodes are linked based on their kappa score level (>0.03). The node size demarcates the enrichment significance of each term. Functionally related groups are partially overlapped. (**b, e**) Histogram of the most enriched biological function specific terms of hypomethylated and hypermethylated genes. The length of bar is percentage of hypomethylated genes in functional terms (GO database), specific gene number are presented beside the bar. (**c, f**) Overview pie chart of the hypomethylated and hypermethylated genes enriched functional groups are presented, the percentage of certain groups were according to GO database analysis. DAVID, the database for annotation, visualization and integrated discovery; GO, gene ontology; and LIMAs, left internal mammary arteries.
