# Supplementary material for: DNA methylome profiling reveals epigenetic regulation of lipoprotein-associated phospholipase A2 in human vulnerable atherosclerotic plaque
Source: Clin Epigenetics. 2021 Aug 21;13:161. doi: 10.1186/s13148-021-01152-z (PMC8379831; doi:10.1186/s13148-021-01152-z)
Supplement: Supplementary file 9 — Additional file 9. Expression of Lp-PLA2 mRNA treated with different concentration of 5-aza-CdR [file 13148_2021_1152_MOESM9_ESM.pdf]

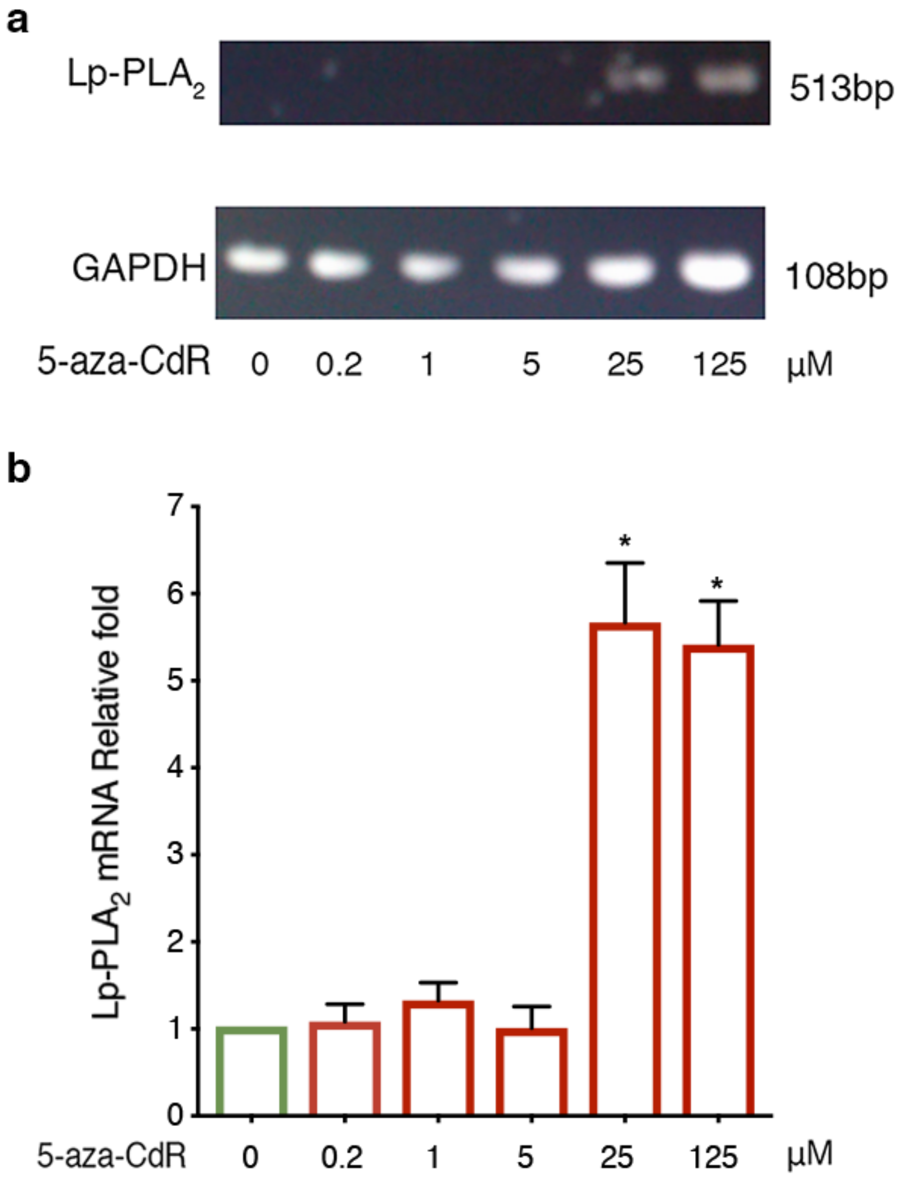

Additional file 9. Expression of Lp-PLA$_2$ mRNA treated with different concentration of 5-aza-CdR. THP-1 cell pretreated with LPS were treated with 5-aza-CdR at 0, 0.2, 1, 5, 25, 125 μM for 48h. The abundance of mRNA is normalized to GAPDH. The levels of Lp-PLA$_2$ mRNA significant increased after treated with 25 and 125 μM 5-aza- CdR. (a) PCR product gel, one representative example of n = 3 different cell batches. (b) Semi-quantitative analysis of PCR, n = 3 different cell batches, mean ± SME, unpaired Student's *t*-test. $^{*}p<0.05$. Lp-PLA$_2$, lipoprotein-associated phospholipase A$_2$; 5-aza-CdR, 5-aza-2'-deoxycytidine; LPS, lipopolysaccharide; and PCR, polymerase chain reaction.
